# Identifying and describing subtypes of spontaneous empathic facial expression production in autistic adults

**DOI:** 10.1186/s11689-022-09451-z

**Published:** 2022-08-01

**Authors:** Jennifer Quinde-Zlibut, Anabil Munshi, Gautam Biswas, Carissa J. Cascio

**Affiliations:** 1 Graduate Program in Neuroscience, Vanderbilt University, Nashville, USA; 2 Frist Center for Autism and Innovation, Vanderbilt University, Nashville, USA; 3 Institute for Software Integrated Systems, Vanderbilt University, Nashville, USA; 4 Department of Psychiatry and Behavioral Sciences, Vanderbilt University Medical Center, Nashville, USA

**Keywords:** Autism spectrum disorder, Facial expression production, Empathy, Nonverbal communication

## Abstract

**Background:**

It is unclear whether atypical patterns of facial expression production metrics in autism reflect the dynamic and nuanced nature of facial expressions across people or a true diagnostic difference. Furthermore, the heterogeneity observed across autism symptomatology suggests a need for more adaptive and personalized social skills programs. Towards this goal, it would be useful to have a more concrete and empirical understanding of the different expressiveness profiles within the autistic population and how they differ from neurotypicals.

**Methods:**

We used automated facial coding and an unsupervised clustering approach to limit inter-individual variability in facial expression production that may have otherwise obscured group differences in previous studies, allowing an “apples-to-apples” comparison between autistic and neurotypical adults. Specifically, we applied k-means clustering to identify subtypes of facial expressiveness in an autism group (*N* = 27) and a neurotypical control group (*N* = 57) separately. The two most stable clusters from these analyses were then further characterized and compared based on their expressiveness and emotive congruence to emotionally charged stimuli.

**Results:**

Our main finding was that a subset of autistic adults in our sample show heightened spontaneous facial expressions irrespective of image valence. We did not find evidence for greater incongruous (i.e., inappropriate) facial expressions in autism. Finally, we found a negative trend between expressiveness and emotion recognition within the autism group.

**Conclusion:**

The results from our previous study on self-reported empathy and current expressivity findings point to a higher degree of facial expressions recruited for emotional resonance in autism that may not always be adaptive (e.g., experiencing similar emotional resonance regardless of valence). These findings also build on previous work indicating that facial expression intensity is not diminished in autism and suggest the need for intervention programs to focus on emotion recognition and social skills in the context of both negative and positive emotions.

## Background

Autism spectrum disorder (ASD; henceforth also referred to as “autism”) is a neurodevelopmental condition characterized by difficulties with socio-emotional communication skills, and engaging in restricted, repetitive patterns of behavior, interests, and activities [1]. Within the socio-emotional domain, the capacity to understand and use nonverbal communication is thought to be central to developing and maintaining healthy social relationships throughout the lifespan [2], as well as facilitate learning and workforce outcomes [3]. Persistent social difficulties translate to difficulties developing and maintaining social relationships throughout adulthood and are associated with depression, anxiety, loneliness, and isolation [4]. Given the pervasiveness and impact of socio-emotional difficulties in autism, many social skills intervention programs are designed to facilitate training in socially relevant nonverbal cue usage, production, and understanding by means of in-person and technology-based paradigms [5].

In-person social skills intervention programs provide structured opportunities to learn and practice social skills and have been shown to improve social metrics like friendship quality, social functioning, and reducing feelings of loneliness in youth and adult autistic groups [6, 7]. Group-based interventions are among the most widely used approaches and yield substantial benefits in self-reported social knowledge, but these gains do not reliably translate to objective laboratory-based measures, or, more importantly, parent/teacher reports [6]. In addition to these limitations, in-person training programs are resource-intensive for both healthcare systems and families, requiring extensive clinical training, administration, and family transportation services. Access to these resources is especially limited given a scarcity of available autism service providers [8] and has been exacerbated by recent COVID-19-related restrictions.

Though they are not yet considered standard of care, emerging automated technology systems supported by machine learning have facilitated the administration and improved the accessibility of autism services like social skills interventions [9]. Supervised and unsupervised machine learning approaches hold promise for predicting outcomes and facilitating the identification of clinical subgroups based on symptom profiles [10]. Current applications for computational methods in autism research include diagnostic methods [11], the analysis of facial expression production [12, 13], and behavioral and physiological signals [14]. Facial expression production and reciprocity are central to important socio-emotional constructs like emotional regulation [15] and the success of social interactions [16]. Within the autistic population, facial expressions are found to be atypical in appearance metrics like social congruence, frequency, or duration [17]. In practice, these differences may lead to negative evaluations from peers and reduce the overall quality of social interactions [18]. Interestingly, previous reports do not suggest that facial expression intensity is affected in autism [19], despite prevalent clinical descriptions of both “flat affect” [20, 21] and “exaggerated” expressions [22, 23]. It is possible however, that in group comparison studies, distinct subgroups of extreme high and low levels of expressivity average one another out and mask differences that are not uniform in direction within the autistic population. Computational approaches such as k-means clustering can help to differentiate this scenario from a true lack of group difference in facial expression intensity.

The heterogeneity observed across autism symptomatology suggests a need for more adaptive and personalized social skill interventions programs. Furthermore, these advancements would benefit from a more concrete and empirical understanding of the different expressiveness profiles within the autistic population and how they differ from neurotypicals (NT) before deploying facial expression production and reception trainings. To this end, we cluster autistic and neurotypical adults separately on the basis of their facial expressions within the socially relevant context of empathy. Empathy has long been considered a sub-domain of the social communication difficulties present in autism [24], but more current evidence suggests a much more nuanced picture [25] given the multifaceted nature of empathy measurement. Relevant domains span psychophysiology, social cognition, and affective response. Thus, our goal was to gain deeper insights on profiles of expressiveness and features of autism across these separate but related domains.

## Methods

Our primary objective for this paper was to explore whether the atypical patterns of facial expression production metrics in autism reflect the dynamic and nuanced nature of facial expressions or a true diagnostic difference. To this end, we collected facial videos during an experimental study, derived a set of automated facial expression features from the videos using the iMotions affect recognition toolkit [26, 27], and applied an exploratory unsupervised learning approach on the feature sets for ASD and NT participants separately to derive interpretable clusters.

### Participants

A total of 84 participants, originally part of a larger study, were included in this analysis. The current sample (*n* = 84) consisted of 27 ASD participants (12 female, 14 male, 1 other) and 57 neurotypical (NT) participants (21 female, 36 male). All participants in this sample were adults between the ages of 18 and 59 years. Participants were pre-screened using the Wechsler Abbreviated Scale of Intelligence Second Edition (WASI-II) [28], with Full Scale IQ (FSIQ) scores ≥ 70. Participants also completed the Social Responsiveness Scale-2 (SRS-2), a self-report questionnaire that measures autistic traits [29]. Autism diagnoses for participants were confirmed by the clinical judgment of a licensed psychologist specializing in the assessment of ASD, supported by research-reliable administration of the Autism Diagnostic Observation Schedule-2 (ADOS-2) [30]. Exclusion criteria for both ASD and NT groups included the presence of other neurological and genetic disorders, non-ASD-related sensory impairments (e.g., uncorrected visual or hearing impairments), and substance/alcohol abuse or dependence during the past 2 years. Furthermore, individuals in the NT group were excluded if they had reported a previous psychiatric history, cognitive or sensory impairment, use of psychotropic medications, or clinically elevated scores on the Social Communication Questionnaire (SCQ) [31]. Individuals with ASD and co-occurring ADHD, anxiety, or depression were included, while those with other recent psychiatric diagnoses within the past 5 years or co-occurring neurogenetic syndromes were excluded. All participants provided informed consent and were compensated $20 per hour of their time following each session. All procedures were approved by the Institutional Review Board for human subjects at Vanderbilt University Medical Center.

### Experimental procedure

We captured participants’ facial expressions while they completed an adapted version of the Multifaceted Empathy Test (MET) [32], a validated multidimensional computer-based task that separates arousal, emotional, and cognitive components of empathy. A full description of the MET can be found in Quinde-Zlibut et al. [25]; briefly, the adapted version presently used includes 32 emotionally charged photographs depicting positive and negative scenarios and is known as the MET-J [33]. When presented with each image, participants were asked to rate their level of arousal, emotional relatedness (emotional empathy), and finally a cognitive empathy (i.e., emotion recognition) multiple choice question. In the present study, the task was designed to be compatible with the iMotions v.6 computer software platform for biosensor integration [26]. The facial expressions of interest for the cluster analysis were recordings from emotional empathy trials where participants viewed an emotional image (of either positive or negative valence) and were asked to answer: “While looking at the picture, how much do your feelings match the boy’s feelings?” Note that while the previous example is for a photograph of a boy, the task included standardized and validated images of males and females of all ages from the International Affective Picture System [34].

### Data collection

**Fig. 1:**
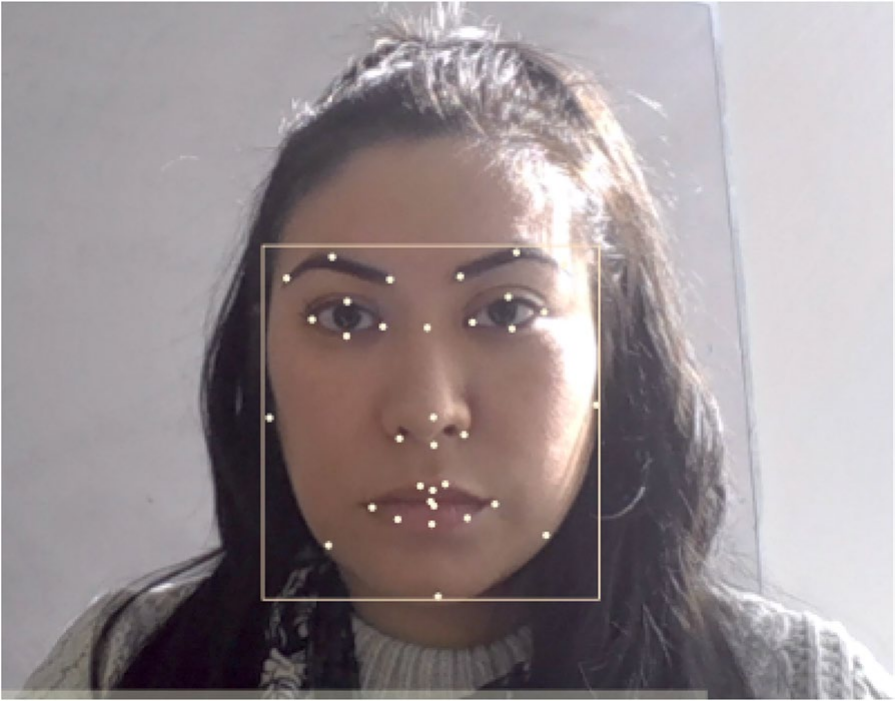
Example of the 33 AffDex detected points around the major landmark facial features. Note that the two points between the lips are really one point that was captured during slight movement

All participants in the MET study worked individually in the same well-illuminated testing room using a webcam-enabled laptop, which facilitated the collection of facial videos. The videos were processed post hoc using the iMotions AffDex SDK. The AffDex engine works by detecting 33 points around major facial landmarks (e.g., eyes, nose, mouth, etc.; Fig. 1), tracking and analyzing them throughout stimuli presentation to identify and classify 20 “facial action units” (AUs; e.g., upper lip raise, outer brow raise, etc.) [27]. Likelihood scores are computed based on the probability that detected AUs are equal to evaluations made by a human rater. Facial expressions or AUs with probabilities below 10% are considered to be of high uncertainty and are thus given likelihood scores of 0. The algorithm, based on Ekman and Friesen’s Emotional Facial Action Coding System (EMFACS) [35], then uses combinations of these facial AUs to compute likelihood scores for the presence of 7 core emotions (joy, anger, fear, disgust, contempt, sadness, and surprise) and summary metrics like *facial engagement/expressiveness* and *emotional valence*. The AffDex channels of interest, derived from the video frames at a frequency of 30Hz, are further defined below:

1. *Engagement/expressiveness*: a general measure of overall facial expressiveness, computed as the average of the highest evidence scores from the upper (brow raise, brow furrow, nose wrinkle) and the lower face region (lip corner depressor, chin raise, lip pucker, lip press, mouth open, lip suck, smile), respectively.
2. *Valence:* a measure of the affective quality of the facial expression, i.e., how positive or negative the associated emotion is. Increased positive valence was determined in AffDex by high likelihood of AUs like *smile* and *cheek raise*, while increased negative valence was determined by high likelihood of AUs like *inner brow raise*, *brow furrow*, *nose wrinkle*, *upper lip raise*, *lip corner depressor*, *chin raise*, *lip press, and lip suck*.

The decision to focus on these summary metrics was made a priori to maximize objectivity and avoid confounds related to assumptions about emotion. Likelihood scores for AUs offer a more concrete and interpretable metric across clinical groups than emotion scores. AffDex scores for AUs typically recruited for expressions of joy (smile) and anger (brow furrow) were found to be significantly correlated to the corresponding EMG metrics (zygomaticus mayor/corrugator supercilii) [36]. We decided against comparing groups based on emotions (e.g., joy, sadness, etc.) because AffDex validation studies suggest that the algorithm’s classification of emotion channels is still too premature for comparative use [37, 38]. Furthermore, the autism-specific FACS and electromyography (EMG) literature is still scant and inconclusive [39, 40] — making it difficult to develop hypotheses regarding how specific AUs are recruited in this population. Derived from individual AUs, summary metrics allow for group comparisons without making assumptions about emotional states underlying specific facial expressions, which may vary by group. Thus, we decided to assess facial expression in terms of overall production (i.e., overall AUs activated in response to stimuli) and appropriateness (i.e., overall congruence of AUs activated in response to stimuli).

### Approach for clustering the ASD and NT groups

#### Feature selection

For each group (NT and ASD), we constructed a set of four features from the data processed through iMotions. The features, listed below, reflect overall levels of facial expressiveness and emotional valence of participants under two different experimental conditions: *(a) When they responded to images evoking positive emotion valence* and *(b) When they responded to images evoking negative emotion valence.* For each participant, we computed the average peak expressiveness and valence scores across trials depicting images of positive and negative emotional valence.

1. Expressiveness (−): average peak expressiveness score for images with negative valence
2. Expressiveness (+): average peak expressiveness score for images with positive valence
3. Valence (−): average peak emotion valence score for images with negative valence
4. Valence (+): average peak emotion valence score for images with positive valence

The *coefficient of variation* (SD/mean) was computed for each constructed feature, as a variance-based feature selection criterion. All four features had coefficients of variation > 20% and were included for clustering. Contrary to values from the engagement channel (which range from 0 to 100), values from the valence channel range from −100 to 100 with negative values indicating negative affect, 0 indicating neutral affect, and positive scores indicating positive affect. Thus, to avoid any potential order-of-magnitude-related feature biases within groups, each feature was *Z*-score standardized across participants. This was done to account for range differences in participants’ responses between the engagement and valence variables and prevent higher values from playing a more decisive role during clustering.

#### K-means clustering

A K-means algorithm was applied on the processed feature set of each group (ASD and NT) using the k-means implementation available in the cluster package [41] in the R environment for statistical computing [42]. K-means is a distance-based algorithm that clusters data points based on how similar they are to one another. Similarity is defined as the Euclidean distance between points such that the lower the distance between the points, the more similar they are. Likewise, the greater the distance, the more dissimilar they are [43]. In practice, the K-means algorithm clusters data points using the following steps:

1. Choice of an optimal value for k clusters: For the present analysis, we used the total within sum of squares (WSS) method. This involves comparing how the WSS changes with increasing number of clusters and identifying the number of clusters associated with the biggest drop in WSS. In our case, the optimal number of clusters determined by this method was *k* = 2 for both the ASD and NT cluster analyses.
2. Random assignment of each data point to an initial cluster from 1 to *K*: This step involves matching each participant with the closest centroid in an *n*-dimensional space where *n* corresponds to the number of features (in this case *n* = 4).
3. Centroid recalculation: After participants are assigned to k clusters, the centroids are recalculated as the mean point of all other points in the group.
4. Cluster stabilization: Steps 2 and 3 are repeated until participants are no longer reallocated to another centroid.

To validate assumptions made about the variance of the distribution of each attribute, the resulting clusters were visually assessed for linear boundaries, and based on their *average silhouette widths*, a measure of how similar each data point is to its own cluster compared to other clusters. Positive silhouette (Si) values indicate appropriately clustered data (the closer to 1, the better the data was assigned). Negative Si values indicate inappropriately clustered data while Si values of 0 indicate that the data point falls between two clusters.

The stability of the resulting clusters was assessed by bootstrap resampling of the data without replacement and computing the Jaccard similarities of the original clusters to the most similar clusters in the resampled data. Jaccard similarity values measure the ratio of points shared between two clusters and the total number of points across both clusters. The mean over the bootstrap distribution of similarity values serves as an index of the stability of the cluster and is henceforth referred to as the Jaccard Index (JI) [44]. Clusters yielding Jaccard Index values < 0.6 are considered to be highly unstable, between 0.6 and 0.75 to be indicative of patterns within the data, ≥ 0.75 to be valid and stable, and ≥ 0.85 to be highly stable [45]. One hundred bootstrap resampling runs were carried out in R using the **clusterboot** function in the *fpc* package [46] and the **kmeansCBI** interface function corresponding to our clustering method.

### Within-group comparisons

Within groups, clusters were compared using a robust, non-parametric effect-size statistic, Cliff’s delta [47, 48] using the *orddom* package [49] in R. Delta does not require any assumptions regarding the shape or spread of two distributions and estimates the probability that a randomly selected observation from one distribution is larger than a randomly selected observation from another distribution, minus the reverse probability. Possible delta (δ) values range from −1 to 1, where values of 0 indicate a complete overlap of groups and values of −1 or 1 indicate that all the values in one group are larger than all the values in the other.

Our variables of interest for this analysis included age, average peak engagement/expressiveness, average emotion congruence, and all the SRS-2 subscales. Average peak engagement/expressiveness was calculated as an average of the expressiveness scores to both negative and positive images. Average congruence was calculated as the average number of instances when a participant’s valence scores matched the emotional valence of the MET images (i.e., when the valence score was greater than 0 and the image was positive, the facial expression was marked as congruent). This metric was calculated across trials as a more intuitive measure of how appropriate participant’s facial expressions were in relation to the valence of the stimuli.

### Between-group comparisons of stable clusters

For the purpose of determining whether there is a true difference in facial expressiveness, we conducted ASD-NT group comparisons on the stable subtypes identified through the separate ASD and NT cluster analyses. Separate robust ANOVAs were computed for average peak engagement and average valence. This analysis was implemented in R using the **bwtrim** function in the *WRS2* package [50]. Briefly, the function adopts a between-within subjects design (i.e., one between-subjects variable and one within-subjects variable) to identify effects based on trimmed means. The trimmed mean discards a specified percentage of values at both ends of a distribution, providing an alternative to the arithmetic mean that is less sensitive to outliers. For both dependent variables, the between-within subjects ANOVA was calculated on the 10% trimmed mean.

### Exploratory analyses

Finally, we ran exploratory correlation tests between average engagement and the emotion recognition scores from the MET-J study [25], ADOS-calibrated severity scores, and SRS subscales (social cognition and social awareness) to better understand the relationship between these variables.

## Results

### K-means clustering

#### ASD cluster analysis

**Fig. 2:**
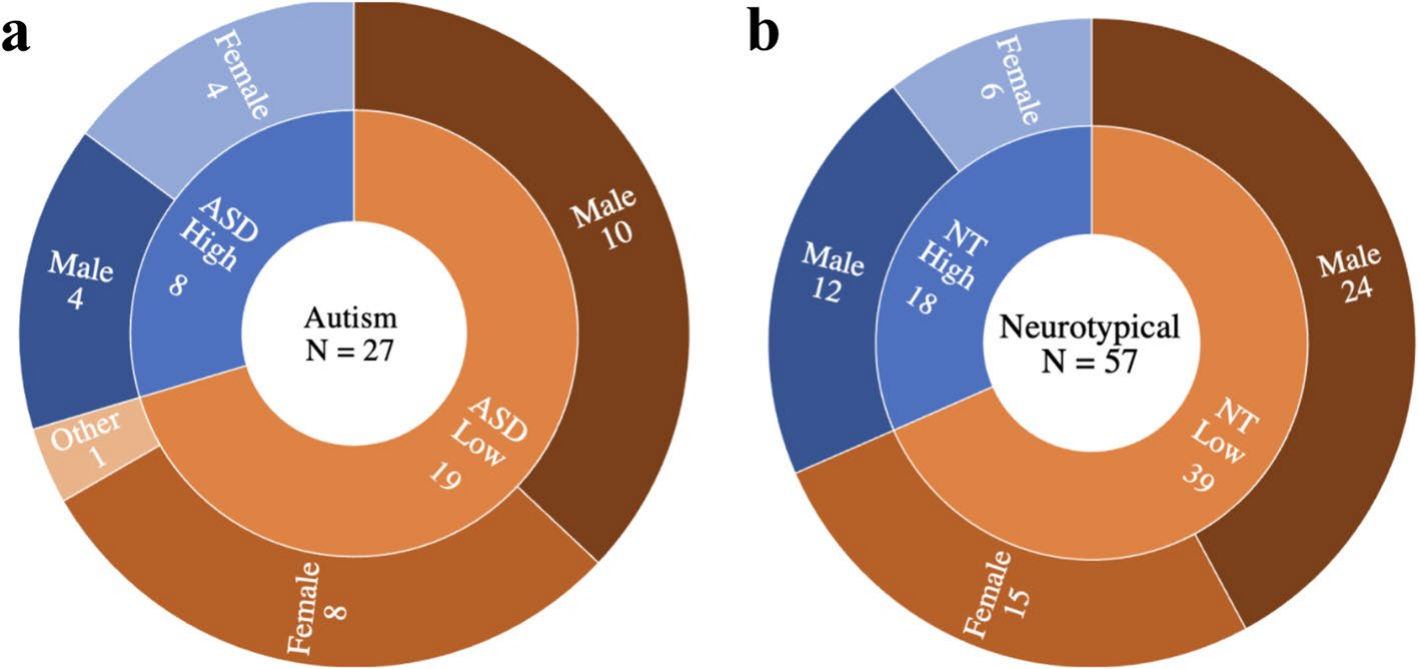
Number of **a** ASD (*n* = 27) and **b** NT adults (*n* = 57) in each cluster grouped by engagement (high, low) and gender (male, female)

**Fig. 3:**
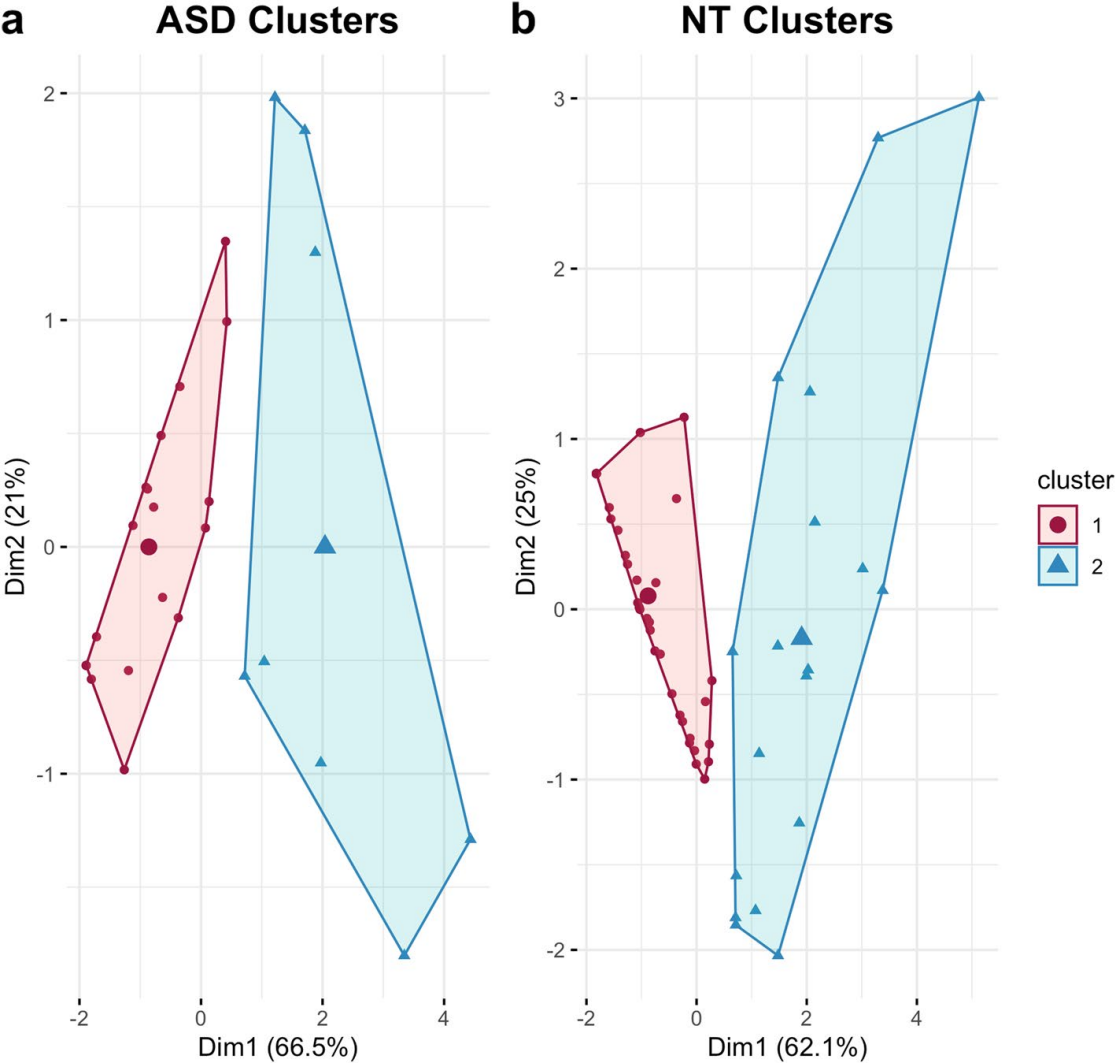
Clusters found in **a** ASD (*N* = 27) and **b** NT adults (*N* = 57). The ASD cluster analysis revealed a larger more stable cluster (cluster 1, *n* = 19) and a smaller, less stable cluster (cluster 2, *n* = 8). The NT cluster analysis revealed a larger more stable cluster (cluster 1, *n* = 39) and a smaller, less stable cluster (cluster 2, *n* = 18)

The k-means model identified two clusters (further characterized in Fig. 2a), within our ASD sample (*N* = 27). The ASD clusters were assessed visually (Fig. 3a), by silhouette (Si) analysis, and the Jaccard Index (JI):

1. Cluster 1 (*n* = 19) with an average Si of 0.56 and JI = 0.886
2. Cluster 2 (*n* = 18) with an average Si of 0.14 and JI = 0.759

**Fig. 4:**
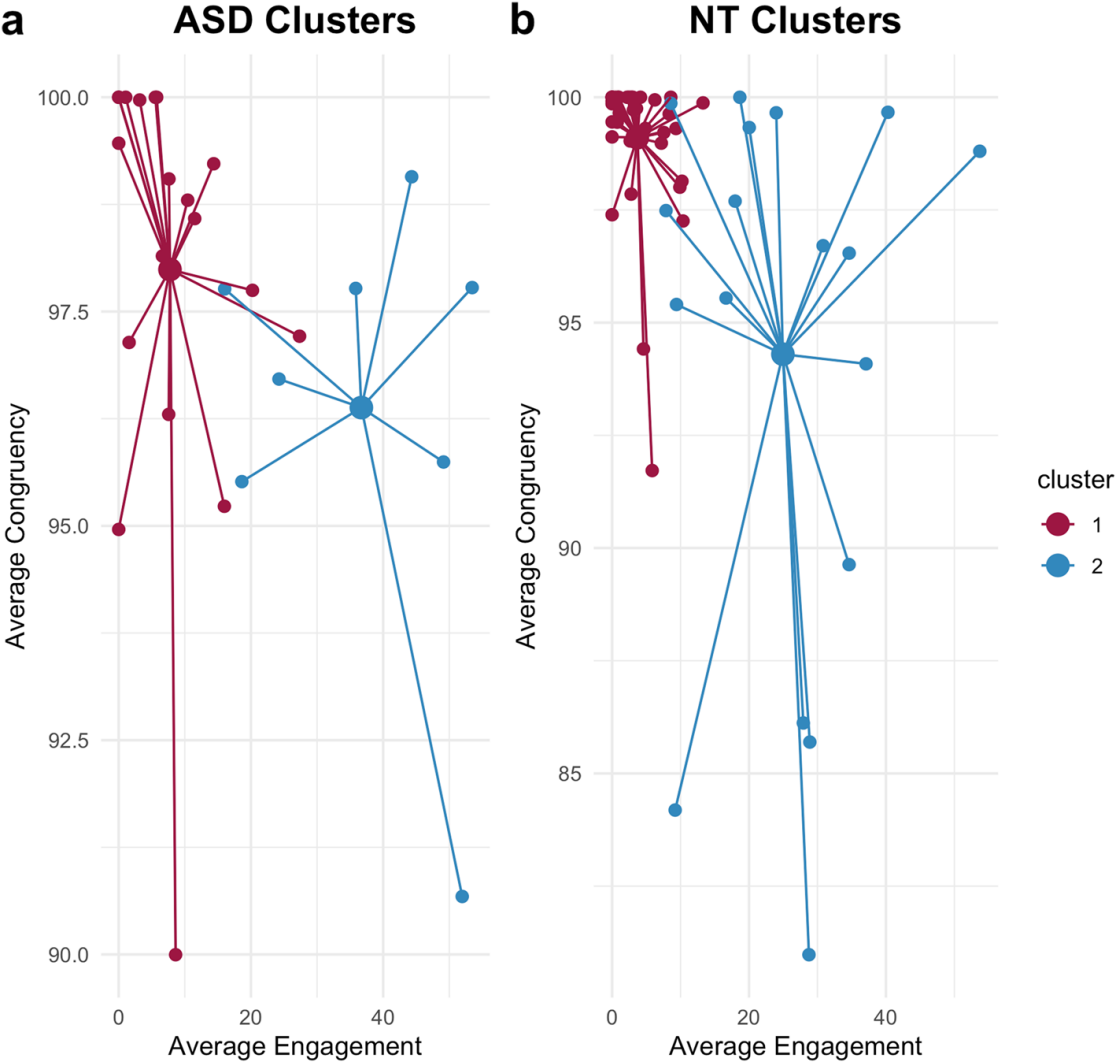
Average congruence and expressiveness/engagement scores found in **a** autistic and **b** neurotypical adult clusters

Subgroup comparisons revealed that cluster 1 differed from cluster 2 in average engagement/expressiveness (*δ* = 0.934, *p* < .001) and average congruence (*δ* = −0.434, *p* = 0.035). Cluster 2 was therefore characterized as a less stable (Si=0.14), more exaggerated group whose facial expressions were less congruent with the stimulus’ emotional valence (Fig. 4a). The clusters did not differ in age or any SRS-2 subscale (Table 1).

**Table 1:** Aggregated statistics on age, facial expressiveness, emotion congruency, and social responsiveness survey (SRS-2) scores for the two ASD clusters

Variable	ASD: cluster 1 (*n* = 19)		ASD: cluster 2 (*n* = 8)		*δ* (95% CI)	*p* value
	Median	SD	Median	SD		
Age (years)	25.97	9.75	22.50	4.36	−0.289 (−0.651, 0.18)	0.203
Expressiveness/engagement	6.61	7.56	40.08	15.29	0.93 (0.731, 0.985)	**0**
Average congruence	98.80	2.53	97.24	2.59	−0.434 (−0.736, 0.012)	**0.035**
SRS-2 (*T*-scores)						
Social awareness	61.00	10.42	57.50	13.73	−0.158 (−0.593, 0.349)	0.548
Social cognition	63.00	10.51	68.00	10.51	0.388 (−0.121, 0.736)	0.103
Social communication	69.00	11.34	64.00	13.99	−0.039 (−0.536, 0.477)	0.891
Social motivation	71.00	11.4	64.00	15.15	−0.132 (−0.611, 0.419)	0.656
Restricted interests and repetitive behavior	73.00	12.36	67.50	16.45	−0.184 (−0.63, 0.353)	0.508

*p* values in bold indicate statistically significant differences between the clusters

#### NT cluster analysis

The k-means model identified two clusters (further characterized in Fig. 2b), within our NT sample (*N* = 57). The NT clusters were assessed visually (Fig. 3b), by silhouette (Si) analysis, and the Jaccard Index (JI):

1. Cluster 1 (*n* = 39) with an average Si of 0.55 and JI = 0.858
2. Cluster 2 (*n* = 18) with an average Si of 0.20 and JI = 0.762

Subgroup comparisons revealed that cluster 1 differed from cluster 2 in average engagement/expressiveness (*δ* = 0.940, *p* < .001) and average congruence (*δ* = −0.625, *p* < .001). Cluster 2 was therefore also characterized as a less stable (Si = 0.20), more exaggerated group whose facial expressions were less congruent with the stimulus’ emotional valence (Fig. 4b). The clusters did not differ in age or any SRS-2 subscale (Table 2).

**Table 2:** Aggregated statistics on age, facial expressiveness, emotion congruency, and social responsiveness survey (SRS-2) scores for the two NT clusters

Variable	NT: cluster 1 (*n* = 39)		NT: cluster 2 (*n* = 18)		*δ* (95% CI)	*p* value
	Median	SD	Median	SD		
Age (years)	29.63	7.68	32.42	13.12	0.099 (−0.281, 0.453)	0.615
Expressiveness/engagement	2.79	3.69	25.95	12.61	0.94 (0.826, 0.98)	**0**
Average congruence	99.72	1.64	96.62	6.16	−0.625 (−0.817, −0.308)	**0**
SRS-2 (*T*-scores)						
Social awareness	44.00	7.86	44.00	7.59	−0.038 (−0.349, 0.281)	0.822
Social cognition	44.00	7.76	44.00	6.26	0.066 (−0.245, 0.364)	0.68
Social communication	45.00	8.01	44.00	7.00	−0.096 (−0.402, 0.229)	0.565
Social motivation	51.00	9.13	52.00	8.64	0.093 (−0.221, 0.39)	0.563
Restricted interests and repetitive behavior	45.00	6.04	47.00	9.93	0.189 (−0.152, 0.49)	0.268

*p* values in bold indicate statistically significant differences between the clusters

The group of NTs that appear on the edge of the convex hull in cluster 1 reflect participants who on average displayed minimal expressivity/engagement in response to emotionally charged stimuli. We refrained from excluding these participants in subsequent analyses because we felt that minimal engagement scores in this NT cluster would be informative compared against the more stable ASD cluster.

### Comparison of the stable ASD and NT clusters

**Fig. 5:**
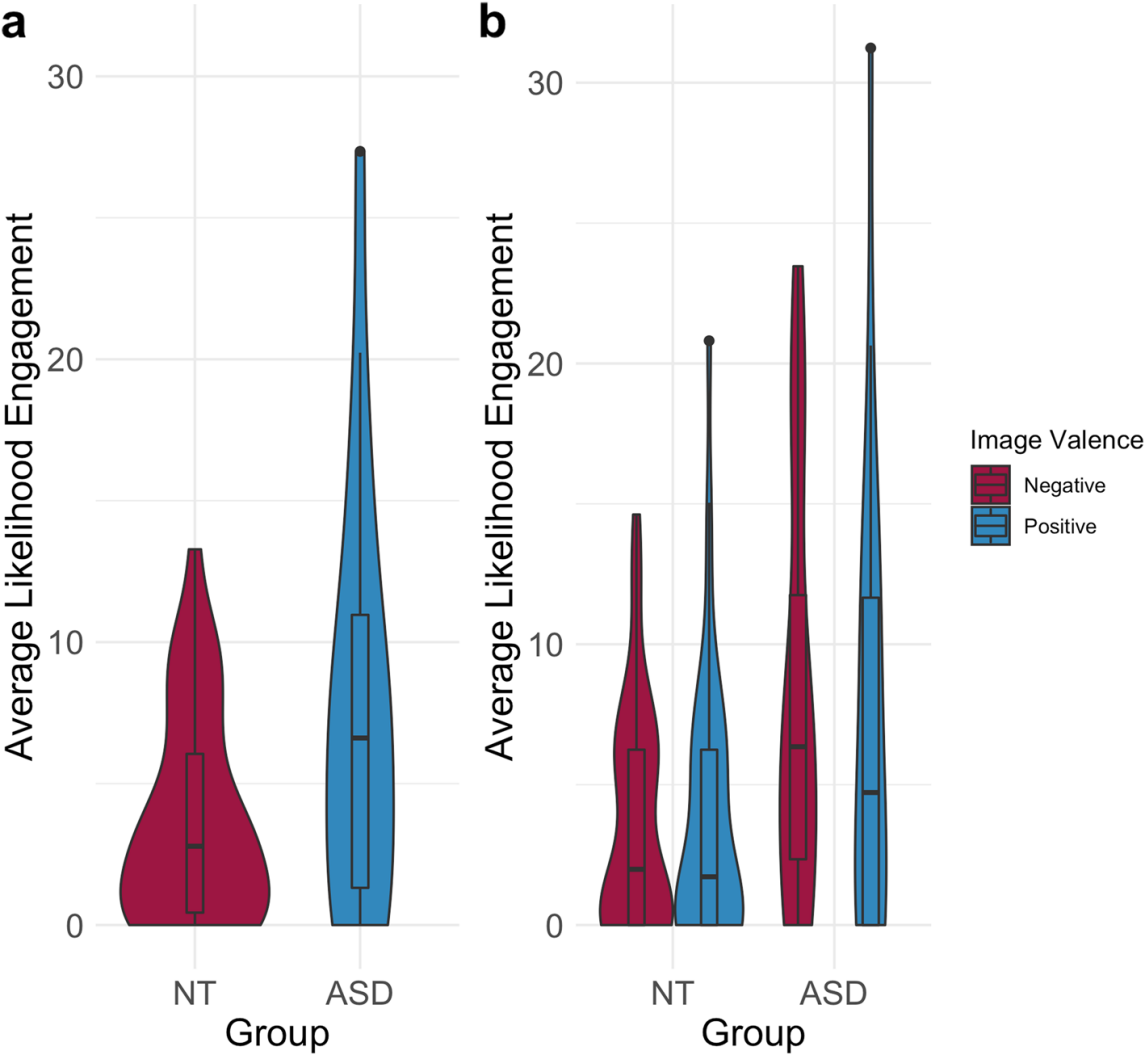
Average expressiveness/engagement scores found **a** between stable groups across images and **b** within stable groups in response to negative and positive images

We selected and compared the two more stable subgroups within our ASD and NT samples to identify whether these differed in average congruence, facial expressiveness, and valence in response to emotional images. We found no group difference in average congruence across images (*δ* = 0.09, *p* > .05). For expressiveness/engagement, the between-within trimmed-means ANOVA revealed a significant difference between groups (*F*(1, 38.58) = 5.02, *p* = .03; Fig 5a), no within-group effect of image valence (*F*(1, 47.59) = 0.54, *p* > .05; Fig 5b), and a non-significant group by image valence interaction (*F*(1, 47.59) = 0.27, *p* > .05). The between-within trimmed-means ANOVA fit for the valence of facial expressions in response to emotional images did not reveal significant between-group differences (*F*(1, 33.44) = 3.78, *p* > .05), within-group differences in response to positive versus negative images (*F*(1, 33.14) = 1.98, *p* > .05), or a group by image valence interaction (*F*(1, 33.14) = 1.39, *p* > .05).

### Exploratory analyses

**Fig. 6:**
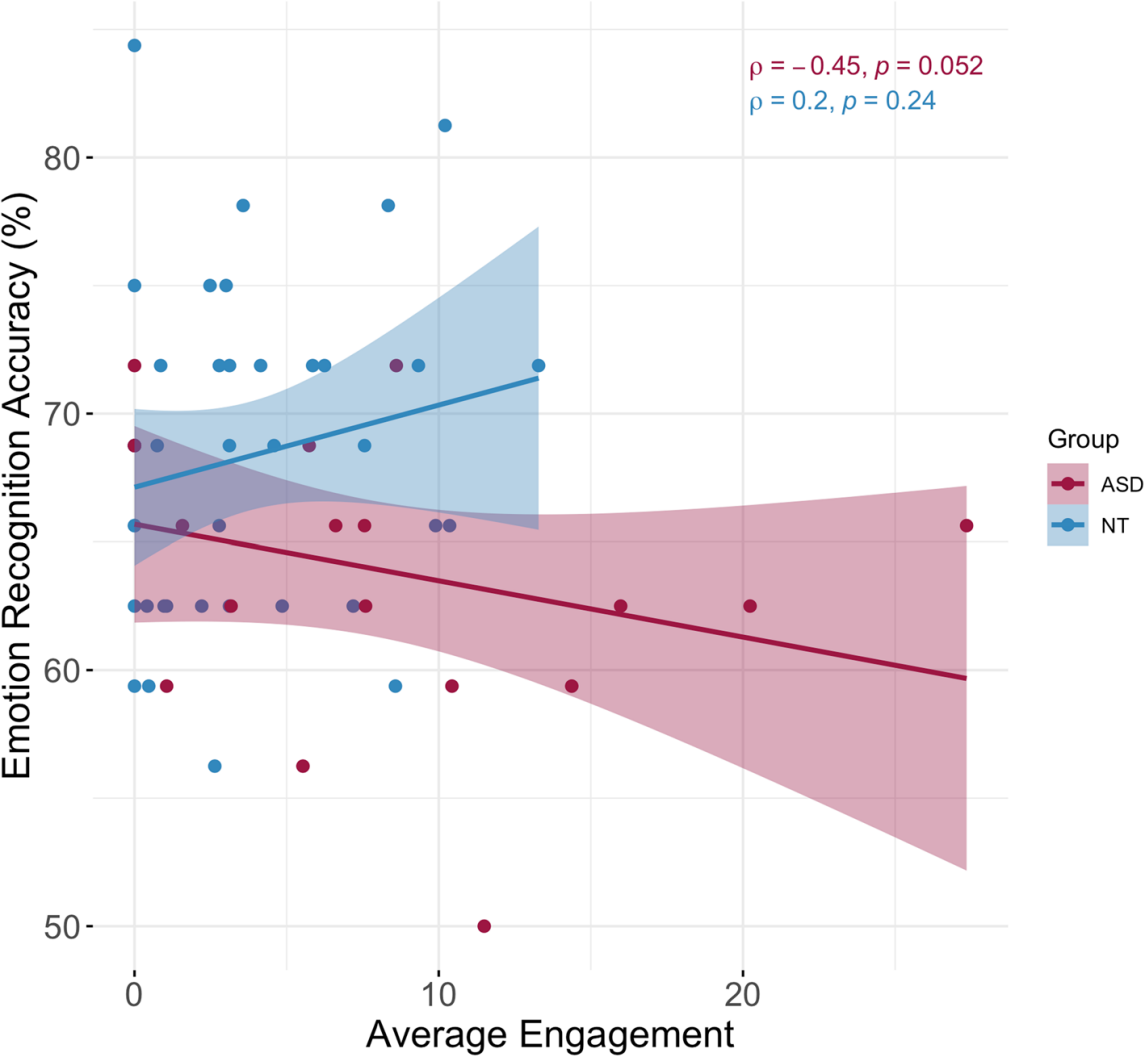
Correlations between emotion recognition accuracy (%) and average engagement across all emotional images for ASD and NT participants in the more stable clusters

Within-group correlations between average engagement and emotion recognition suggest that in ASD, higher expressivity/engagement is associated with poorer performance in the emotion recognition component of the MET-J study (*ρ* = −0.45, *p* = 0.05). We observed a weaker and opposite trend in the NT group of increased emotion recognition performance with more expressivity/engagement (*ρ* = 0.20, *p* > 0.05; Fig. 6). Average expressivity/engagement was not correlated to ADOS-calibrated severity scores (*ρ* = −0.13, *p* > 0.05), the social cognition (*ρ* = 0.11, *p* > 0.05), or social awareness (*ρ* = 0.06, *p* > 0.05) subscales of the SRS-2 in our stable autism sample.

## Discussion

In this study, a primary goal was to use computational approaches to address discrepancies in the literature on spontaneous empathic facial expressions in autistic adults. Facial expression as a means of registration and communication of emotion is a highly nuanced behavioral phenomenon characterized by high inter-individual variability [51] and strong developmental effects [52]. The literature is further complicated by the usage of a variety of research methods, with drastic differences in the method of eliciting facial expressions (ranging from explicitly asking participants to produce a facial expression to eliciting spontaneous expressions with a non-social (e.g., a foul odor) or a social (e.g., another face making an expression) stimulus). Studies also differ in the method of measuring facial expressions (ranging from coding by observers who may or may not have formal training in facial coding [35] to electromyography of facial muscles, or automated algorithms for coding facial action). Thus, methodological and individual variability has presented a challenge to a clear understanding of how facial expression production differs in autism. A recent meta-analysis [17] found that, across various approaches, autistic people on average appear to differ on the quality and frequency of facial expressions, but are largely similar to neurotypicals in the intensity and timing of facial expressions. However, given that the studies in those analyses included a range of the aforementioned variations and noted moderating effects of individual factors, there is still a considerable lack of clarity on the effect of autism on spontaneous empathic facial expressions specifically, which are more likely to relate to empathy than elicited/requested expressions or spontaneous expressions to non-social stimuli.

For this reason, we focused on spontaneous facial expressions to images depicting an emotional face—a variant of facial mimicry. We restricted our sample to adults and used automated facial coding to capture participants’ spontaneous facial expressions when viewing images of other people in emotionally charged situations. These data were then subjected to clustering analyses to isolate reliable subgroups based on overall levels of facial expressiveness or engagement. We found that both autistic and non-autistic adults could be separated into two clusters: a larger cluster with relatively lower overall expressivity and more within-cluster homogeneity in the recruitment of spontaneous facial expressions, and a smaller cluster that exhibited higher expressivity overall but with significant variability between individuals in the cluster.

To limit the potential for inter-individual variability in this nuanced behavior to obscure meaningful differences, we used only the larger and more stable cluster in each group for subsequent group analyses. Our three primary findings in this subset were surprising. First, the autistic group showed higher overall facial engagement or expressivity in response to the emotional images, without significant effects of image valence or an interaction between image valence and group. Second, counter to predictions based on the appropriateness of facial expressions to the social situation, the two groups did not differ on congruency (the extent to which the participant’s facial expression matched that in the image) or in their valence experience in response to either positive or negative images. Finally, in the autistic adults, higher levels of facial expressivity were negatively related to accuracy in the emotion recognition task, while a weaker trend was opposite in direction for the neurotypical group. We will explore each of these findings below.

Our primary finding that a sample of autistic adults, from which a small cluster of variable but highly expressive individuals was already removed, still showed higher levels of facial expressivity in response to emotional images. This finding is consistent with reports of intact facial mimicry in autism [53] and more intense spontaneous facial expressions in adolescents with autism during non-social contexts [54], as well as with the findings of a large meta-analysis [17] demonstrating that autistic individuals do not show diminished intensity of facial expressions across contexts. Indeed, we find that in the context of spontaneous response to emotionally charged images, autistic adults on average respond with more facial expressivity. In our previous work using the same stimuli [25], both groups experienced greater self-reported emotional resonance (i.e., emotional empathy) to positive versus negative images. There was also significantly less differentiation between self-reported emotional resonance to positive versus negative emotional images in our autistic group. For this reason, we expected interactions between group and valence in spontaneous facial expressions, which are thought to reflect emotional resonance/empathy. However, we did not detect any interactions, suggesting that these spontaneous facial expressions may represent more than simply a reflection of emotional resonance.

The presence of subgroups and group differences based on intensity and congruence of facial expressions in both the neurotypical and autistic samples without the accompanying differences in the congruence or appropriateness of facial expressions between the two more stable clusters suggests a possible role for individual differences in the affective and sensorimotor aspects of facial expression production. Motor programs to produce a spontaneous facial expression in response to an emotional image may be initiated as expected, suggesting intact feedforward input from amygdala to facial motor circuitry [55]. However, in autistic adults, the end result of executing this program is an amplified facial expression, which could reflect altered use of sensory feedback from facial skin and muscle to both facial motor and affective brain regions.

While we did not find significant relations between clinical variables such as ADOS-calibrated severity scores or SRS subscale score and our main outcome measure of facial engagement, we noted an interesting dichotomy in the way that facial engagement associates with accuracy of emotion recognition on the MET-J. For the autistic group, higher facial engagement/expressivity was related to lower emotion recognition (i.e., cognitive empathy). One interpretation of this unexpected finding is that increased facial expressivity is an effect, rather than a cause, of social difficulty. As adults engage in a task that is challenging to them—identifying the emotions of another person—increased facial engagement could arise from increased concentration or worry [56]. An alternative interpretation is that amplified facial expressions may contribute to social difficulty. Previous studies suggest that these two interpretations may be mutually related; adults with autism are more tolerant of exaggerated emotional facial expressions than neurotypical adults, and this is thought to reflect a rule-based strategy employed by autistic adults when learning to interpret emotional facial expressions [57], a process that may involve amplified facial mirroring in an attempt to learn the associations.

### Limitations and future directions

Our finding of equivalent valence in the more stable clusters does not preclude a subset of individuals characterized by inappropriate or incongruent facial expressions, as is commonly described clinically in a minority of people on the spectrum. Indeed, the smaller and less stable cluster in our ASD sample may represent this subset of the autistic population. A limitation of this study is the small sample size that prevented us from further defining this subgroup. Our small sample size also warrants well-powered follow-up studies to confirm the present results.

Other limitations of the study include the use of static stimuli rather than dynamic or interactive social stimuli; thus, future work should consider alternative paradigms that more closely align with real-world social situations that elicit spontaneous facial expressions. We based our decision to derive within-group clusters on preliminary analyses demonstrating poor cluster assignment when the ASD-NT data were pooled. We believe these preliminary and current findings point to the highly variable nature of facial expression use across our sample regardless of diagnostic status.

Algorithm-based metrics of emotion, trained on people without autism, are likely to lead to results that are not applicable to autism. We address this concern by limiting our choice of metrics to overall engagement and valence (distinct from AffDex-classified emotions like “joy” or “surprise”) which are solely based on facial actions and compared against the probability that they are equal to scores from human coders. Future complementary studies should include complete FACS coding assessments of separate clusters to identify AU-specific differences. In this scenario, the combination of AFC, clustering, and FACS could reduce the amount of FACS coding hours considerably.

Currently, socio-emotional autism literature is dominated by top-down paradigms that do not address the inherent reciprocity in dyadic interactions [58–60], thereby limiting our understanding of social phenomena to a stereotypical “norm”. Indeed, the presence of a smaller, more expressively variable cluster in our NT sample suggests that the expressivity patterns observed in the smaller ASD cluster may not be so “atypical.” Both the autism and social skill intervention fields will benefit from future work that explores socio-affective phenomena from this less biased framework. Future studies should also examine this phenomenon in child and/or adolescent samples and individuals with co-occurring intellectual disability to better understand the influence of development and cognitive ability.

## Conclusion

In this study, our main finding was that a subset of autistic adults in our sample show heightened spontaneous facial expressions regardless of image valence. We used automated facial coding and a clustering approach to limit inter-individual variability that may have otherwise obscured group differences in previous studies, allowing an “apples-to-apples” comparison between autistic and neurotypical adults. We did not find evidence for greater incongruous (i.e., inappropriate) facial expressions in autism. Taken together, our self-report and expressivity findings point to a higher degree of facial expressions recruited for emotional resonance in autism that may not always be adaptive (e.g., experiencing similar emotional resonance regardless of valence). Finally, these findings build on previous reports indicating that facial expression intensity is not diminished in autism and suggest the need for intervention programs to focus on emotion recognition and social skills in the context of both negative and positive emotions.

## Data Availability

The dataset used and/or analyzed during the current study are available from the corresponding author on reasonable request.

## Abbreviations

ASD: Autism spectrum disorder
NT: Neurotypical
COVID: Coronavirus disease
WASI: Wechsler Abbreviated Scale of Intelligence
ADOS: Autism Diagnostic Observation Schedule
ADHD: Attention deficit hyperactivity disorder
MET: Multifaceted Empathy Task
EMFACS: Emotional Facial Action Coding System
AU: Action unit
SD: Standard deviation
WSS: Within sum of squares
SRS: Social Responsiveness Scale
